# High-resolution dissection of photosystem II electron transport reveals differential response to water deficit and heat stress in isolation and combination in pearl millet [*Pennisetum glaucum* (L.) R. Br.]

**DOI:** 10.3389/fpls.2022.892676

**Published:** 2022-08-12

**Authors:** Arun K. Shanker, Sushma Amirineni, Divya Bhanu, S. K. Yadav, N. Jyothilakshmi, M. Vanaja, Jainender Singh, B. Sarkar, M. Maheswari, V. K. Singh

**Affiliations:** Indian Council of Agricultural Research (ICAR)-Central Research Institute for Dryland Agriculture, Hyderabad, India

**Keywords:** phenomenological fluxes, oxygen evolving complex (OEC), heat stress, water deficit stress, photosystem II

## Abstract

Heat and Water Deficit Stress (WDS) tend to impede and restrict the efficiency of photosynthesis, chlorophyll fluorescence, and maximum photochemical quantum yield in plants based on their characteristic ability to interfere with the electron transport system in photosystem II. Dissection of the electron transport pathway in Photosystem II (PSII) under water deficit and Heat Stress (HS) can be insightful in gaining knowledge on the various attributes of the photosynthetic performance of a plant. We attempt a high-resolution dissection of electron transport in PSII with studies on chlorophyll a fast fluorescence kinetics and non-photochemical quenching (NPQ) as a response to and recovery from these stresses in pearl millet [*Pennisetum glaucum* (L.) R. Br.] in isolation and combination. In this study, we bring out the mechanisms by which both heat and water stress, in isolation and in combination, affect the photosynthetic electron transport in Photosystem II. Our results indicate that oxygen evolution complex (OEC) damage is the primary effect of heat stress and is not seen with the same intensity in the water-stressed plants. Low exciton absorption flux in heat stress and combined stress was seen due to OEC damage, and this caused an electron transport traffic jam in the donor side of PS II. Both the specific energy flux model and the phenomenological flux model developed from the derived values in our study show that water deficit stress in combination with heat stress has a much stronger effect than the stresses in isolation on the overall electron transport pathway of the PS II in pearl millet plants.

## Introduction

The projected models of climate change and many other future scenarios predict that there will be an increased probability of long dry spells, temperature extremes, and heat spells in various regions of the world (Murari and Ghosh, 2019; Wainwright et al., 2021). Over the last half a century, there has been an increase in the percentage of surface land that has been impacted by continuous heat and dry spells (Pascale et al., 2016; Rothlisberger and Martius, 2019). Heat and water deficit, in isolation and in combination, substantially contribute to alterations in the physiological processes in plants in general and in specific; these stresses considerably impact photosynthesis, causing the decline in growth of the crops and dry matter yield (Balfagon et al., 2020; Parvathi et al., 2020). Both these stresses can have a significant deleterious effect on plants, particularly when they occur in tandem or simultaneously. The effect of these two stresses has not been studied in detail with specific reference to electron transport dynamics of the photosystem II in environmentally relevant conditions.

To evolve climate-ready cultivars of various crops, we need a clear understanding of the mechanism by which crops are affected by stresses, such as heat and water deficit. Such studies will help us identify traits that give specific adaptive advantages to crops to overcome these stresses, and this can be taken forward by breeders to evolve climate-ready cultivars (Scheben et al., 2016; Shanker and Shanker, 2016).

Crop diversification and the cultivation of new crops that have the inherent ability to tolerate stresses are some of the ways to tackle climate change. In this context, millets, in general, and pearl millet [*Pennisetum glaucum* (L.) R. Br.], in particular, can be a good choice as an alternative crop as it is predominantly cultivated on marginal drylands that have frequent dry spells and heat waves occurring in tandem. It ranks sixth in the list of important food crops of the world and is grown in over 30 million hectares all over the world, mainly in the tropics (Sun et al., 2020). The drought-tolerant characteristics of pearl millet are due to its well-formed root system, which allows mining for water and nutrients under water-limiting conditions (Soni, 2020). The flowering phenology, tillering behavior, and dry matter accumulation at various phenophases of pearl millet are well-adjusted to confer adaptation to adverse environmental conditions (Pearson, 1975; Choudhary et al., 2020).

This hardy nature of the crop (Walter and Morio, 2005; Soni, 2020) makes it very suitable to explore the underlying mechanism of heat and water deficit tolerance; due to these reasons, it can be termed as a model crop for various mechanistic studies on abiotic stress tolerance at a greenhouse and the field level. Specifically, in this context, the study of its photosystem II dynamics under water deficit and Heat Stress (HS) can be insightful in gaining knowledge of the various aspects of the photosynthetic performance of this crop, which forms the basis of dry matter accumulation and yield. To the best of our understanding and knowledge, there have been no in-depth studies on chlorophyll a fluorescence kinetics and the electron transport pathway of Photosystem II in this crop as affected by heat and water deficit, although our group have worked on the Potassium (K) fertilization effects on Photosystem II in pearl millet and other crops (Srinivasarao et al., 2016). It is possible that a clue to the pearl millet's phenomenal ability to tolerate heat and drought and recover from short- to medium-term stress lies in its ability to regulate and adjust its photosynthetic system under adverse conditions apart from various other genetic and metabolic mechanisms. Water relations in plants are regulated by various factors, and understanding the physiological mechanisms is important to tackle climate-related threats to crop production (Li et al., 2022). In addition to heat, low temperatures also affect photosystem II electron transport. Low temperature seriously depresses the growth of wheat through inhibition of photosynthesis, while earlier cold priming may enhance the tolerance of plants to subsequent low-temperature stress (Li et al., 2014). Similarly, continuous and fluctuating light regimes are also known to affect Photosystem II electron transport (Ferroni et al., 2020).

Stress due to water deficit and HS is closely related in its nature of incidence and, also, in the way it affects the plants. Drought can last for several weeks, and heat can be experienced by the crops as waves or hot spells and is more often accompanied by water deficit (Zhu L. et al., 2020). This gives rise to cross stress in crops, which is a combination of these stresses, and the effects of it are less studied in controlled conditions. The water deficit and heat interactive effects can induce complex physiological responses, specifically in the photosynthetic apparatus (Eustis et al., 2020). Gas exchange is known to be affected by both drought (Lang et al., 2018; Chaturvedi et al., 2019; Liu et al., 2019) and heat (Haworth et al., 2018; Poudyal et al., 2018; Chovancek et al., 2021), with a sharp decline in the rate of photosynthesis, stomatal conductance, and the rate of transpiration due to drought and reduction in the photosynthetic rate and an increase in the stomatal conductance and the transpiration rate due to heat. Here, in our study, we attempt to dissect the response to and recovery from these stresses in isolation and in combination that may be an acclimation phenomenon or, simply, a response to the stress stimuli. Either way, the in-depth study of the photosystem II electron transport chain will give us an opportunity to gain insight into the mechanism by which water deficit stress and heat stress act on the plants.

Heat and water-deficit stress tend to hinder and change photosynthetic efficiency, chlorophyll fluorescence, and maximum photochemical quantum yield in plants based on their characteristic ability to interfere with the electron transport system in photosystem II (Botyanszka et al., 2020; Garcia-Parra et al., 2020). Research focusing on these specific effects and understanding the underlying mechanism is lacking, and there is a knowledge gap here that needs to be addressed. Insights gained here can take us several steps forward in countering stress and effectively developing climate-ready cultivars. The chlorophyll a fluorescence OJIP transient (OJIP transient) method to study the photosystem II dynamics and electron transport perturbations under stress is a widely accepted method and is acclaimed as a robust method (Stirbet, 2011; Zivcak et al., 2015; Romero et al., 2020; Tsimilli-Michael, 2020). This has been a preferred method of study under drought stress in plants (Guha et al., 2013; Cicek et al., 2019) and under heat stress (Mathur et al., 2018; Zhou et al., 2018), whereas few studies have been done under combined drought and heat stress and recovery (Zhu et al., 2021).

Drought and heat are known to affect every step of the electron transport pathway in photosystem II. Drought has been reported to affect the PS II in several ways like stabilizing primary and the secondary quinone acceptor QA and QB, respectively (Leverne and Krieger-Liszkay, 2021), reduction in the performance index (PI_ABS_) (Bano et al., 2021), increase in the net rate of closure reaction centers (Li et al., 2020). Drought stress also has been shown to increase absorption flux per cross-section (ABS/CSo) and decrease the phenomenological energy fluxes for TRo/CSo, ETo/CSo, RC/CSo (Zhao et al., 2019). Heat stress in plants has shown to affect photosystem II in a drastic way (Toth et al., 2007; Yan et al., 2013; Haworth et al., 2018); it has been shown to decrease maximum quantum yield for primary PSII photochemistry (ϕPo = Fv/Fm) and induce changes in both energy and phenomenological fluxes (Gupta, 2019). Heat stress is mainly implicated in the disruption of the oxygen-evolving complex (OEC) in PSII and, further, inhibition of electron transport to the acceptor side of PS II (Gupta, 2020; Hu et al., 2020; Dogru, 2021; Zha et al., 2021). Pigments regulate photochemistry of Photosystem II (PSII), light energy absorption, photon capture and subsequent trapping excitation energy, and conversion of excitation energy into flow of electrons. The presence of higher amounts and specific ratios of these pigments offers protection against stress-related damage to the photosystem II (Berne et al., 2018; Ruban, 2018; Demmig-Adams and Adams, 2019; Giossi et al., 2020).

We hypothesize that heat stress and water deficit stress in isolation and in combination can have an impact on the photosystem II dynamics, electron transport chain, and pigment concentrations in the leaf and gas exchange characteristics of a plant. We also hypothesize that, during stress, specific strategies that can confer adaptive advantages can be employed by plants in terms of changes in photosystem II dynamics, and this can possibly help the plants acquire new homeostasis, which can be a protective adaptation.

## Materials and methods

### Plant material and growth conditions

The study was conducted at ICAR—Central Research Institute for Dryland Agriculture (ICAR-CRIDA) for 4 years (2017–2020) on pearl millet [*Pennisetum glaucum* (L.) R. Br.]. The varieties used in the study were—ICMH 356 and 86M86; they were procured from International Crops Research Institute for the Semi-Arid Tropics (ICRISAT), Hyderabad. The parents of ICMH356 are lines—ICMA 88004 and ICMR 356 ICRISAT 1993; the crop has 90-day duration to maturity and harvest; the crop has a medium stature, with conical ear heads that are thick and semi compact; the anthers are yellow with bold, ovate-shape grains that are yellow brown in color. The mean yield of the crop is 2,300–2,500 kg.ha^−1^. The parents of the variety 86M86 are lines M128F × M138R; the mean yield performance of this variety in various soil and agronomic conditions is good due to its lodging resistance, robust stem and root system, and stay-green quality, with a yield of 2,700–2,900 kg.ha^−1^. This variety also comes to maturity at 90 Days After Sowing (DAS).

Growing conditions of pearl millet plants consisted of 52 pots grown in open ambient atmospheric conditions wherein the mean length of day was 12.5 h. The treatments consisted of control, Water-Deficit Stress (WDS), heat stress (HS), and Combined Stress (CS), which is water-deficit stress in combination with heat stress. The number of replications was 5 for each treatment, and a set of 52 pots was maintained for each of varieties ICMH 356 and 86M86. Twelve pots were kept aside as buffer plants.

The experiment was taken up in 2017 and repeated every year for 4 years in 2018, 2019, and 2020. The sowing of the crop was taken up between the second fortnight of August and the first fortnight of September in each year; the plants were harvested between the last week of November and the 1st week of December when the crop was at about 103 DAS at maturity. The pots were irrigated with tap water. The well-watered pots were irrigated every second day till the soil was saturated with water.

**Figure 1:**
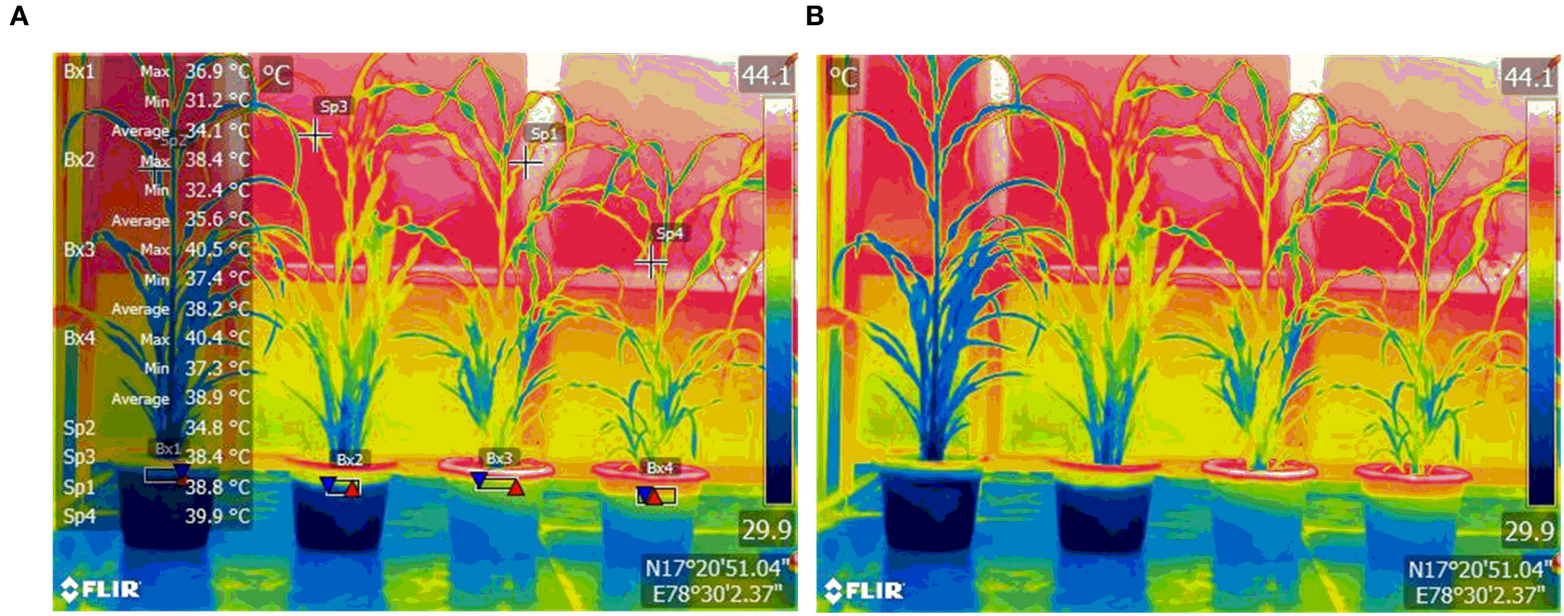
Thermal image of pearl millet plants, Control (extreme left), and treated plants—Water-Deficit Stress, Heat Stress, and Water Deficit + Heat Stress (from left to right), with temperature annotations **(A)** and without temperature annotations **(B)**. Images were taken with Infra Red camera FLIR E-95 at a 4.9-meter distance from the plants. Temperatures of the plants are given as Maximum, Minimum, and mean. Boxes (Bx) show the temperature of the plant at the base, and Spots (Sp) show temperatures at specific spots.

Imposition of WDS was done on both the WDS treatments by withholding of irrigation from the 43 DAS till 54 DAS for a total of 12 days. Imposition of HS was done in such a way that it coincided with the start of stress in the WDS treatments. HS was imposed at 48 DAS plants in heat, and HS in combination with WDS treatments by transfer of pots to phenomics greenhouse enclosure. The ambient temperature conditions in the green house were maintained at 4–5-degree centigrade higher than the ambient temperature in the open conditions. The mean maximum temperature and minimum temperature in the open atmospheric conditions were 32.7 and 22.1°C, respectively. The mean maximum temperature and the minimum temperature in the heat treatment conditions were 37.1 and 27.7°C, respectively. Thermal imaging was conducted after 5 days of HS treatment imposition with Infra-Red camera FLIR E-95 fitted with autofocus, which is laser assisted and has an on-screen area measurement; the temperature points measured are 161,472, with wide temperature ranges. Thermal images were captured at approximately 5-m distance from the plant with emissivity of the thermal camera set at 0.95. Rewatering was done in the stress treatments on the 12th day after treatment imposition. Rewatering was continued for 4 days after which recovery observations were taken on 58 DAS. The thermal image taken with Infra-Red camera FLIR E-95 at 4.9-meter distance from the pearl millet plants is shown in Figure 1.

### Soil moisture and water status

Continuous monitoring of soil moisture content in the pots and Relative Water Content (RWC) in the leaves was done throughout the treatment imposition period. Soil moisture at different depths (at 7.6-, 12-, and 20-cm depth) was estimated by the Time Domain Refractometry (TDR) method using shaft-mounted soil moisture probe TDR 350—Field-Scout, Spectrum Technologies Inc., Aurora, USA. The calibration of the probe was done with a standard graph from gravimetrically obtained values of soil moisture at the different depths of the soil studied plotted with the Volumetric Water Content (VWC) obtained from the instrument. Leaf RWC was determined according to the Barrs and Weatherley (1962) method. The soil moisture content at 7.6-, 12-, and 20-cm depth of soil in the pots and leaf RWC is given in Supplementary Table 1.

### Gas exchange parameters

The gas exchange parameter photosynthetic rate (A), stomatal conductance (gs), and the transpiration rate (Tr) were measured on the second leaf from the apex, which were expanded fully in each of the pots. The response curves A/Ci for changes in the photosynthetic rate with the concentration of CO_2_ internally in the leaf were generated a using Li-6400 portable infrared gas analyzer (LICOR, Inc., Lincoln, NB) according to Manter and Kerrigan (2004). Gas exchange observations on stress treatments were taken on 55 DAS, and recovery observations were taken on 64 DAS 9 days after the rewatering schedule was started.

### Chlorophyll fluorescence parameters

The chlorophyll fluorescence induction kinetics and another full set of chlorophyll fluorescence parameters were measured using Handy PEA, Hansatech Instruments Ltd., Narborough Road, Pentney, King's Lynn, Norfolk PE32 1JL England. Further calculations on flux ratios of the photosystem II, namely, —(i) Maximum yield of primary photochemistry (ΦPo), (ii) Efficiency with which the exciton, which has been trapped, can move an electron into the electron transport chain beyond QA-(Ψo), (iii) Quantum yield of electron transport (Φ) and the specific energy fluxes (per reaction center) (i) Absorption (Abs/RC), (ii) Trapping (TRo/RC), (iii) Dissipation (DIo/CS), and (iv) Electron transport (ETo/RC) was done with the PEA Plus software.

Dark adaption of the leaves was standardized for each of the experiments, and the correct time for dark adaptation was estimated by the following method: A Leaf clip was placed on the leaf surface and allowed to dark adaptation of leaves for 5 min; the values of Fv/Fm were recorded by the instrument set at full intensity for 1 s. The sample was then readapted for 10 min, and Fv/Fm was recorded; the procedure was repeated for 15, 20, and 25 min of readaptation. The dark adaptation time after which there was no increase in the Fv/Fm reading was taken as the standard time for dark adaptation of the samples. We found that there was no increase in the Fv/Fm values after 15 min of dark adaptation in all the experiments; hence, we took 15 min as the standard time for dark adaptation in all the experiments.

The optimum light intensity for measurement of chlorophyll fluorescence parameters was arrived at by placing 10-leaf clips in ten leaves and measuring Fv/Fm, following 15-min dark adaptation, which was arrived at as described above. Measurements were made on each leaf at increasing light levels between 0 and 3,500 μmol m^−2^ s^−1^. The saturation point of light intensity above which there was no further increase of Fv/Fm was taken as the optimum light intensity for the measurement of chlorophyll fluorescence parameters. The saturating intensity was found to be 3,000 μmol m^−2^ s^−1^.

All the chlorophyll fluorescence parameters were measured at ambient temperature (in HS and CS treatments the ambient temperature was the concerned heat treatment temperature), with the instrument with high-time resolution (10 μs). The dark adaptation period was 15 min.

### Oxygen evolving complex measurements

The OJIP test was utilized for estimating the ${\text{O}}_{2}^{-}$evolving centers in the control and stress treatments. The method adopted was from Srivastava et al. (1997), Strasser (1997), Lazar and Pospisil (1999), and Lazar (1999), who reported that the K step, at about 300 ms, is due to the deactivation of OEC. The fraction of ${\text{O}}_{2}^{-}$ evolving centers in comparison with the control was calculated using the following formula:

W–Variable fluorescence intensity normalized to the J-step (W = Ft–F0)/(FJ–F0)
W_K_ Amplitude of the K-step (WK = VK–VJ)
Fraction of Oxygen-Evolving Complexes expressed as percentage reduction over control
[1–VK/VJ] of stressed plants/[1–VK/VJ] where *V*_K_ is the variable fluorescence at
300 μs = (F_300μs_-F_50μs_)/(F_M_-F_50μs_), and *V*_J_ is the variable fluorescence at
2 ms = (F_2ms_-FS_0μs_)/(F_M_-F_S0μs_), respectively.

The complete list of Chlorophyll fluorescence parameters recorded and calculated is given in Table 1.

**Table 1:** Definitions, explanations, and calculations of the JIP test parameters used in the present study (adopted from Strasser et al. (2004)).

**Data extracted from the recorded fluorescence transient O-J-I-P**	
F_t_	Fluorescence attimetafter onset of actinic illumination
F_50μ*S*_ or F_20μ*s*_	Minimal reliable recorded fluorescence, at 50 μs with the PEA-or 20μs with the Handy-PEA-fluorimeter
F_100μ*S*_	Fluorescence at 100 μs
F_300μ*S*_	Fluorescence at 300 μs
F_*J*_ = F_2ms_	Fluorescence at the J-step (2 ms) of O-J-I-P
F_I_ = F_2ms_	Fluorescence at the I-step (30 ms) of O-J-I-P
F_P_ = F_M_)	Maximal recorded (=maximal possible) fluorescence, at the peak P of O-J-I-P
^t^F_M_	time(in ms) to reach maximal fluorescence F_M_
Area	Total complementary area between fluorescence induction curve and F = F_M_
**Fluorescence parameters derived from the extracted data**	
F_0_ ≅F_50μS_ or ≅ F_20μS_	Minimal fluorescence, when all PS II RCs are open (at *t* = 0)
F_M_ = F_P_	Maximal fluorescence, when all PS II RCs are closed
F_ν_ = F_t_-F_0_	Variable fluorescence at time t
F_V_ **=** F_M_-F_0_	Maximal variable fluorescence
Vt = (F_t_-F_0_)/(F_M_-F_0_)	Relative variable fluorescence at time t
V_J_ = (F_J_-F_0_)/(F_M_-F_0_)	Relative variable fluorescence at the J-step
Wt **=** (F_t_-F_0_)/(F_J_-F_0_)	Ratio of variable fluorescence F_ν_ to the amplitude F_J_-F_0_
W_E, 100μS_ = 1–(1–W_300μS_)^1/5^	W at 100 μs of a simulated exponential fluorescence transient corresponding to the sample in the absence of grouping (i.e., no connectivity between PS II units)
M_0_ = (ΔV/Δt)_0_ = 4 (F_300μS_-F_0_)/(F_M_-F_0_)	Approximated initial slope (in ms^−1^) of the fluorescence transient V = f(t)
S_m_ = (Area)/(F_M_-F_0_)	Normalized total complementary area above the O-J-I-P transient (reflecting multiple-turnover Q_A_ reduction events)
S_S_ = V_J_/M_0_	Normalized total complementary area corresponding only to the O-J phase (reflecting single-turnover Q_A_ reduction events)
N = S_m_/S_S_ = S_m_M_0_ (1/V_J_)	Turnover number: number of Q_A_ reduction events between time 0 and ^t^ F_M_
V_av_ = 1–(S_m_/^t^F_M_)	Average relative variable fluorescence from time 0 to ^t^F_M_
**Specific energy fluxes per Q**_**A**_ **-reducing PSII reaction center—RC**	
ABS/RC =M_0_ (1/V_J_)(1/ϕ_P0_)	Absorption flux per RC
TR_0_/RC = M_0_ (1/V_J_)	Trapped energy flux per RC (at *t* = 0)
ET_0_/RC = M_0_ (1/V_J_) ψ_0_	Electron transport flux per RC (at *t* = 0)
DI_0_/RC (ABS/RC)–(TR_0_/RC)	Dissipated energy flux per RC (at *t* = 0)
**Yields or flux ratios**	
ϕ_P0_ = TR_0_/ABS = [1–(F_0_/F_M_)]	Maximum quantum yield of primary photochemistry (at *t* = 0)
ψ_0_ = ET_0_/TR_0_ = (1–V_J_)	Probability (at *t* = 0) that a trapped exciton moves an electron into the electron transport chain beyond ${\text{Q}}_{\text{A}}^{-}$
ϕ_E0_ = ET_0_/ABS = [1–(F_0_/F_M_)] ψ_0_	Quantum yield of electron transport (at *t* = 0)
ϕ_D0_ = 1–ϕ_P0_ = (F_0_/F_M_ )	Quantum yield (at *t* = 0) of energy dissipation
ϕ_P0_ = ϕ_P0_ (1 V_av_) = ϕ_P0_ (S_m_/^t^F_M_)	Average (from time 0 to ^t^F_M_) quantum yield of primary photochemistry
**Phenomenological energy fluxes Lper excited cross section CS**	
ABS/CS_X_	Absorption flux per CS “_X_” (subscript “x” can be “Ch1,” “0,” or “M;” see below)
ABS/CS_ch1_	Absorption flux per CS, determined by reflectance measurements (a measure of Ch1/ CS)
ABS/CS_0_ ≈ F_0_	Absorption flux per CS, approximated by F_0_
ABS/CS_M_ ≈ F_M_	Absorption flux per CS, approximated by F_M_
TR_0_/CS_X_ = ϕ_P0_(ABS/CS_X_)	Trapped energy flux per CS (at *t* = 0)
ET_0_/CS_X_ = ϕ_E0_(ABS/CS_X_)	Electron transport flux per CS (at *t* = 0)
DI_0_/CS_X_ = (ABS/CS_X_)–(TR_0_/CS_X_)	Dissipated energy flux per CS (at *t* = 0)
**Density of reaction centers**	
RC/CS_X_ = ϕ_P0_(V_J_/M_0_)(ABS/CS_X_)	Density of RCs (Q_A_-reducing PSII reaction centers)
**Performance indexes (PI) at** ***t*** **=** **0**	
${\text{PI}}_{\text{ABS}}\equiv \frac{RC}{ABS}\;.\frac{\varphi_{P0}}{1-\varphi_{P0}}\;.\frac{\psi_{0}}{1-\psi_{0}}$	Performance index on absorption basis
${\text{PI}}_{\text{CS}}\equiv \frac{RC}{CS_{X}}\;.\frac{\varphi_{P0}}{1-\varphi_{P0}}\;.\frac{\psi_{0}}{1-\psi_{0}}$	Performance index on cross-section basis
**Driving forces (logarithms of performance indexes at** ***t*** **=** **0)**	
DF_ABS_ ≡ log (PI_ABS_) = $log\;\frac{RC}{ABS}\;+\;log\;\frac{\varphi_{P0}}{1-\varphi_{P0}}+\;log\;\frac{\psi_{0}}{1-\psi_{0}}$	Driving force on absorption basis
DF_CS_ ≡ log (PI_CS_) = $log\;\frac{ABS}{CS_{X}}\;+\;log\;\left(PIABS\right)$	Driving force on cross-section basis
**Overall grouping or connectivity probability**	
$P_{2G}\;=\;\frac{\left(W_{E,100us\;}W_{100us}\right)F_{0}}{W_{100us}\;\left(\;1\;W_{E,100us\;}V_{J}\;\right)\;V_{J}F_{V}}$	Grouping probability taking in account all possible ways of energetic communication of neighboring PSII core antennae

### Non-photochemical quenching

Non-photochemical quenching was measured using a portable fluorometer (FluorPen FP 100; Photon Systems Instruments; Drasov, Czech Republic). The measurements were done according to standardized protocol developed in our earlier study (Srinivasarao et al., 2016). The youngest leaves that were fully developed were taken for the measurement of chlorophyll fluorescence parameters. Dark adaptation of the leaf with leaf clips provided by the manufacturer of the instrument was done for 30 min before starting the measurement. The adaxial leaf surface was taken for measurements and was done two times at adjacent points in the leaf. The data were pooled data from 10 measurements from five plants with two-point measurements for each leaf, and one leaf per plant for each treatment and control were taken for analysis.

Predefined protocol for the measurement was used with two phases, namely, light and dark recovery, lasting 60 and 88 s, respectively. The number of pulses during the light phase was 5 s, and the number of pulses during the dark recovery phase was 3 s, with the first pulse at 7 and 11 s in the light and dark recovery phase, respectively. The pulse interval was 12 s in the light phase and 26 s in the dark-recovery phase.

### Carotenoids and chlorophyll content of leaf tissue

Carotenoids content and chlorophyll content expressed as fresh weight in the plant leaf were measured (mgg^−1^ of fresh weight) by following the procedure according to Arnon (1949) and Wellburn (1994). We followed the methods we had described in our earlier work (Srinivasarao et al., 2016).

Chla (mg g–^1^) = 0.0127 A663–0.00269 A645
Chlb (mg g–^1^) = 0.0029 A663–0.00468 A645
Total Chl (mg g–^1^) = 0.0202 A663 + 0.00802 A645
Carotenes and xanthophylls = [(1,000.A470)–(3.27.Chl a)–(1.04.Chl b)]/229,

Where Chl a—chlorophyll a, in mgl, Chl b—chlorophyll b, in mg/l, A470—sample absorbance at 470 nm.

### Statistical analysis

Five replicates were taken for all the parameters from each set of the experiments (*n* = 20). The mean values ± S.E. are given in all the tables and figures. Statistical analysis of data was performed using the Windows-based SPSS program (IBM Corp. Released 2017. IBM SPSS Statistics for Windows, Version 25.0. Armonk, NY: IBM Corp.). The General Linear Model (GLM) procedure was used for the ANOVA and to determine the statistical significance of treatment effects. The Tukey's test was used as a *post-hoc* test to test significance between means (Haynes, 2013). The OJIP curve, the J-step-normalized curve, and the NPQ curve were reported as means of n = 20 for variety ICMH 356.

## Results

### Gas exchange parameters

The rate of photosynthesis, stomatal conductance, and the transpiration rate of control and stress-treated plants and their values in the recovery stage of the treatments is shown in Table 2. There was a significant decrease in both the varieties, with 9.3-μmol CO_2_ m^−2^ s^−1^ decrease in the photosynthetic rate in the WDS treatments as compared to control as against a 13 CO_2_ m^−2^ s^−1^ decrease in the HS treatment and 15.6 CO_2_ m^−2^ s^−1^ decrease in water deficit, in combination with HS treatment. The WDS recovery was much better than the recovery of HS and HS in combination with WDS treatments with regard to the photosynthetic rates of these recovery plants.

**Table 2:** The photosynthetic rate and gas exchange parameters in two varieties of pearl millet under water-deficit stress and heat stress in isolation and in combination and after recovery from stress.

**Treatment**	**Photosynthetic rate**		**Stomatal conductance**		**Transpiration rate**	
	**(**μ**mol CO**_**2**_ **m**^**−2**^ **s**^**−1**^**)**		**[mol (H**_**2**_**O) m**^**−2**^ **s**^**−1**^**]**		**[mmol (H**_**2**_**O) m**^**−2**^ **s**^**−1**^**]**	
	**86M86**	**ICMH356**	**86M86**	**ICMH356**	**86M86**	**ICMH356**
Control	31.9 (± 0.154)a	32.4 (± 0.108)a	0.52 (±0.013)a	0.56 (± 0.048)a	6.78 (± 0.161)	6.98 (± 0.217)a
Water deficit stress	22.6 (± 0.160)b	22.5 (± 0.188)b	0.21 (± 0.072)b	0.23 (± 0.077)b	3.97 (± 0.185)	3.78 (± 0.225)b
Heat stress	18.7 (±0.110)c	19.6 (± 0.193)c	0.20 (± 0.055)b	0.17 (± 0.091)c	3.99 (± 0.161)	4.01 (± 0.187)b
Water deficit + heat stress	16.7 (± 0.202)c	16.4 (± 0.040)d	0.20 (± 0.060)b	0.19 (± 0.045)b	3.43 (± 0.198)	3.27 (± 0.186)c
Control recovery	31.2 (± 0.267)a	31.4 (± 0.303)a	0.52 (± 0.061)a	0.54 (± 0.049)a	6.81 (± 0.189)	6.87 (± 0.177)a
Water deficit stress recovery	28.5 (± 0.313)a	26.0 (± 0.440)b	0.50 (± 0.053)a	0.50 (± 0.096)a	6.04 (± 0.212)	6.56 (± 0.174)a
Heat stress recovery	23.6 (± 0.170)b	23.1 (± 0.028)c	0.49 (± 0.060)a	0.49 (± 0.068)a	5.99 (± 0.160)	5.65 (± 0.209)b
Water deficit + heat stress recovery	22.4 (± 0.086)b	22.8 (± 0.086)c	0.45 (± 0.075)b	0.49 (± 0.047)a	5.45 (± 0.159)	5.78 (± 0.210)c

*Figures within parentheses are SE, and letters indicate significance at 0.05, and different letters indicate a significant difference between treatments*.

There was a difference of the 3–4-μmol CO_2_ m^−2^ s^−1^ carbon dioxide assimilation rate in the recovered plants when compared with their respective stress-treated plants. Stomatal conductance decreased uniformly and significantly in the water stress and water + HS treatment in both the varieties with no significant difference seen between treatments. Stomatal conductance did not show any significant difference under HS treatments in both varieties. The recovery in stomatal conductance was up to the mark of their respective treated plants with slightly less values seen in the recovered plants as compared to control. The transpiration rate followed a similar trend as seen in stomatal conductance, except for the variety ICMH356 that showed a significant difference among treatments wherein water deficit + HS was lesser than the rest of the stress treatments. Unlike stomatal conductance, the transpiration rate was significantly higher under HS-only treatments in both varieties.

### Chlorophyll a fluorescence transient kinetics

**Figure 2:**
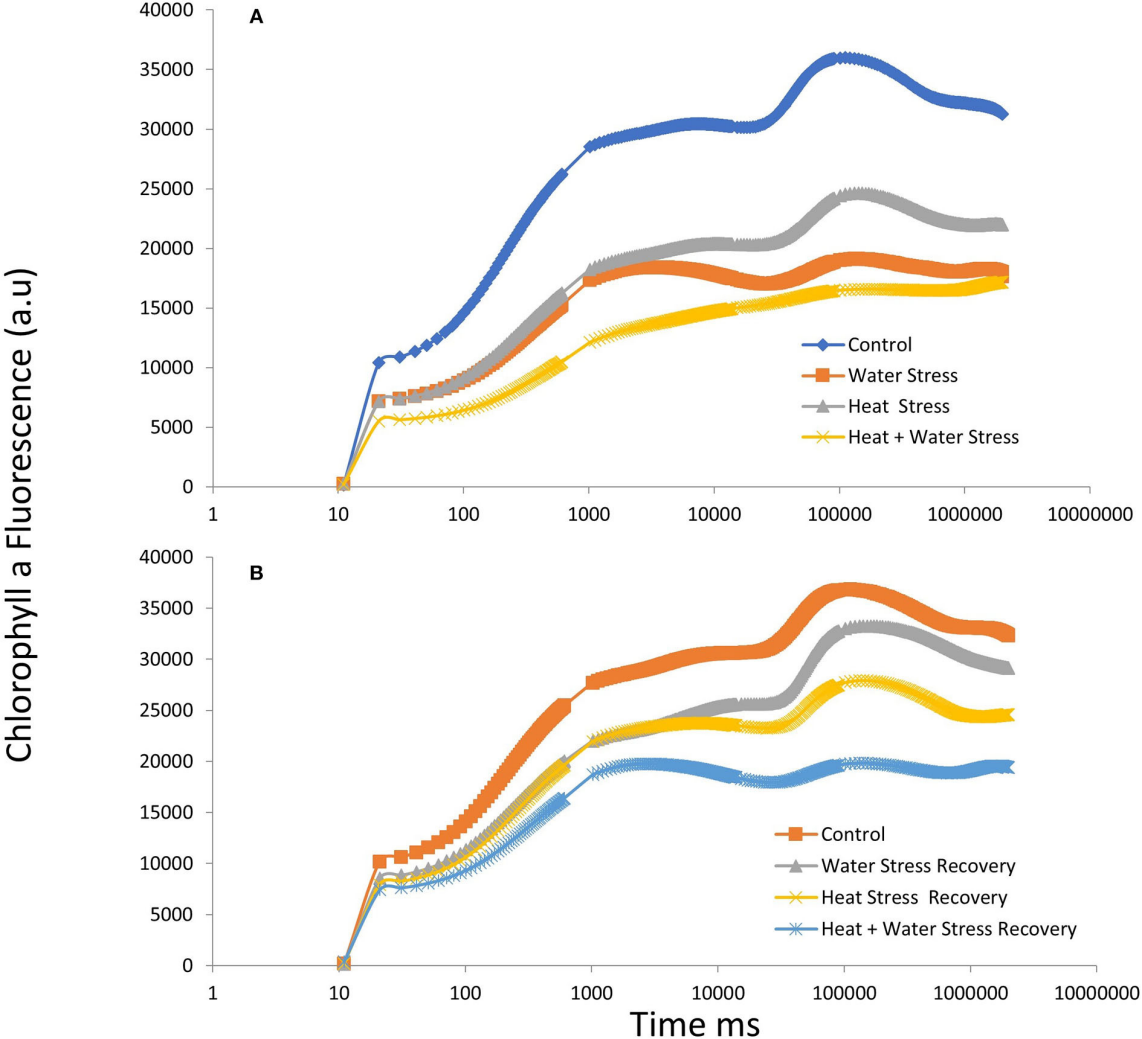
OJIP chlorophyll a fluorescence transients in ICMH356 variety of pearl millet under water-deficit stress and heat stress in isolation and in combination **(A)** and at recovery from stress **(B)**.

The fast fluorescence kinetics in terms of fluorescence yield rose from the normal initial phase F0 (O step) to the maximum fluorescence Fm (P step), with the anticipated transitional Steps J and I in a typical fashion in the control plants. Kautsky curves and the OJIP data plot representing the Chlorophyll a fluorescence transient kinetics in the stressed plants and during recovery in variety ICMH356 are shown in Figures 2A,B, respectively. It was observed that there was a perceptible dip in the curves in stressed plants with heat, combined heat, and WDS, showing a high reduction in the curve. The stressed plants in comparison to control exhibited a reduction in the magnitude of a signal that caused a delay in the J, P, and I steps. The control plants exhibited a classical OJIP curve against the stressed plants where there was an evident deviation from the classical shape. This was more pronounced in the combined stressed plants. The relative variable fluorescence (Vt) deviated clearly from the typical OJIP curve. The water-deficit stressed plants and the heat-stressed plants regained, to a large extent, the curve shape after recovery; on the other hand, the combined stressed plants did not regain the shape completely after recovery.

### K-step

**Figure 3:**
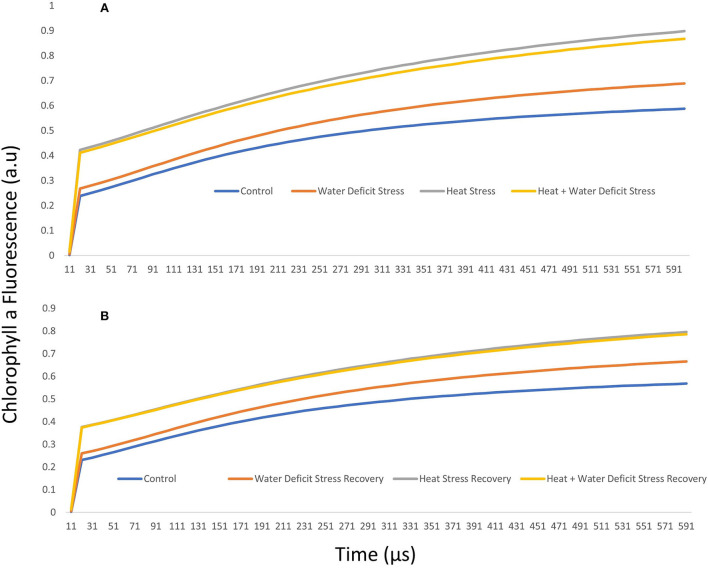
J-step normalized fluorescence (W) in ICMH356 variety of pearl millet under water-deficit stress and heat stress in isolation and in combination **(A)** and at recovery from stress **(B)**.

The normalized intensity of variable fluorescence to the J-step (W = Ft–F0)/(FJ–F0) for stress treatment and after recovery in ICMH 356 is given in Figures 3A,B, respectively. The K step is not easily identifiable from the typical OJIP curve, so we used a better form of a plot to identify the possible K-step, with the plot of relative variable fluorescence W = Vt/VJ at 300 μs. The level was stable in control and recovery plants at approximately 0.50 and increased perceptibility in the heat-stressed plants.

**Figure 4:**
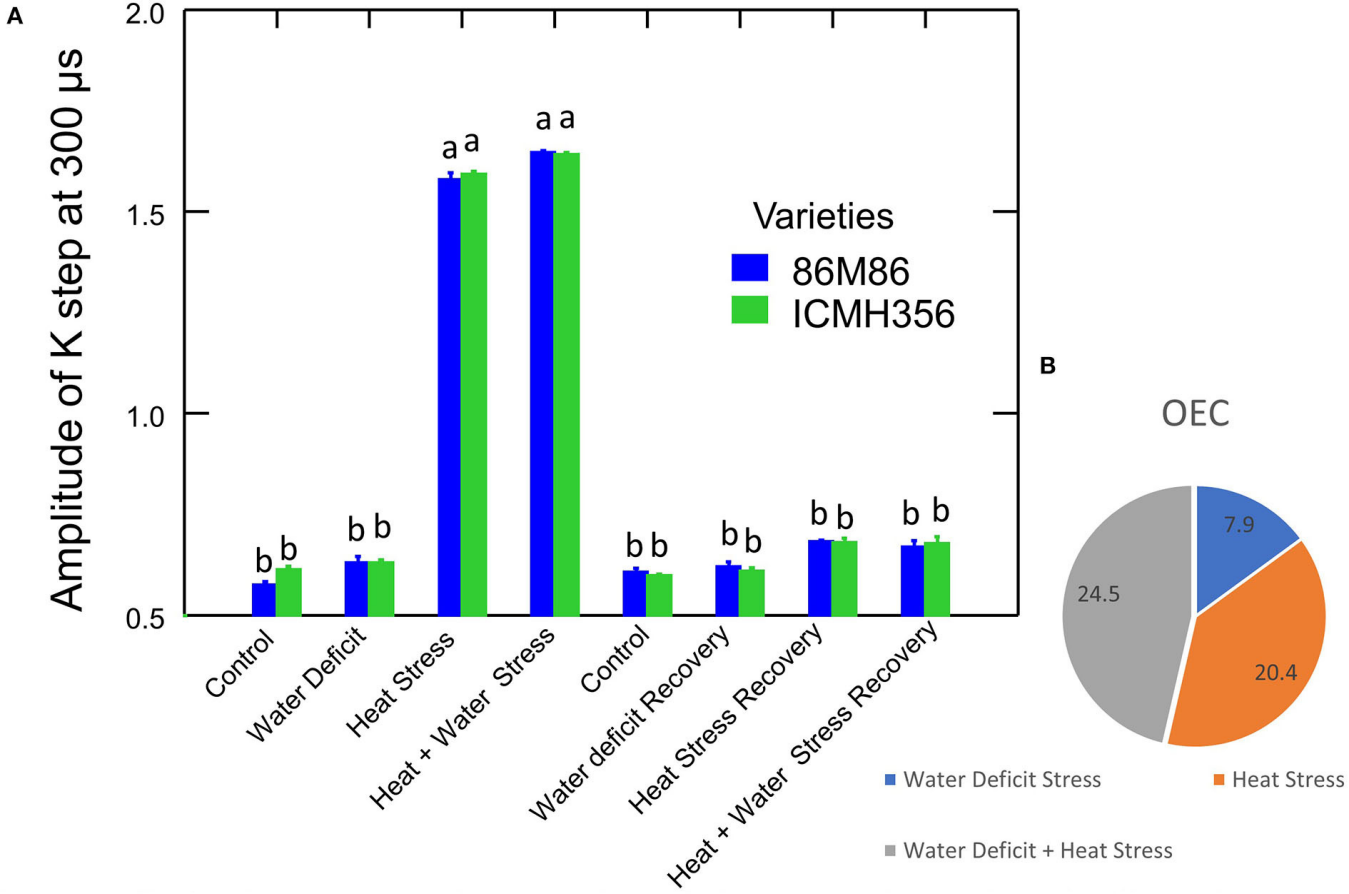
Amplitude of K-step 300 μs (WK = VK/VJ value) in two varieties of pearl millet under recovery from water-deficit stress and heat stress in isolation and in combination. The bar represents SE. Letters indicate significance at 0.05, and different letters indicate significant difference between treatments **(A)** Oxygen-Evolving Complex (OEC) reduced in stress treatments over control [(1–VK/VJ) stress/control] expressed in percentage **(B)**.

It was seen that the plants under WDS treatment and HS treatment in isolation and in combination showed higher chlorophyll a fluorescence arbitrary unit than control. HS and HS in combination with WDS showed more than about a 0.2-higher chlorophyll a fluorescence arbitrary unit than only water deficit and control treatment at the 300-μs mark. This tends to level off as the polyphasic graph enters the next phase of the OJIP curve. In order to discern the so-called K-step more clearly, a better method of plotting the graph with variable fluorescence WK = VK/VJ at time 300 μs was used and shown in Figure 4A. It is seen here in the graph that HS treatment and HS treatment in combination with WDS showed significantly higher values of WK. The amplitude of the K step as quantified by WK in HS and HS in combination with WDS was more than 1 unit than in control and WDS. After recovery, the evident K step amplitude was not seen in all the stress-recovered plants. Both the varieties, 86M86 and ICMH356, exhibited a similar pattern in the response to stresses and recovery, with no significant differences observed between them. Figure 4B shows the Oxygen-Evolving Complex (OEC) of stress expressed as percentage reduction over control as a Pie chart in the stressed treatment as means of both the varieties of pearl millet. Among the treatments, WDS showed a 7.9 per cent decrease in OEC over control, and HS showed a 20.4% decrease over control and the highest reduction over control of 24. Approximately, 5% in the OEC was seen in HS in combination with WDS treatment.

### Non-photochemical quenching and pigments

**Figure 5:**
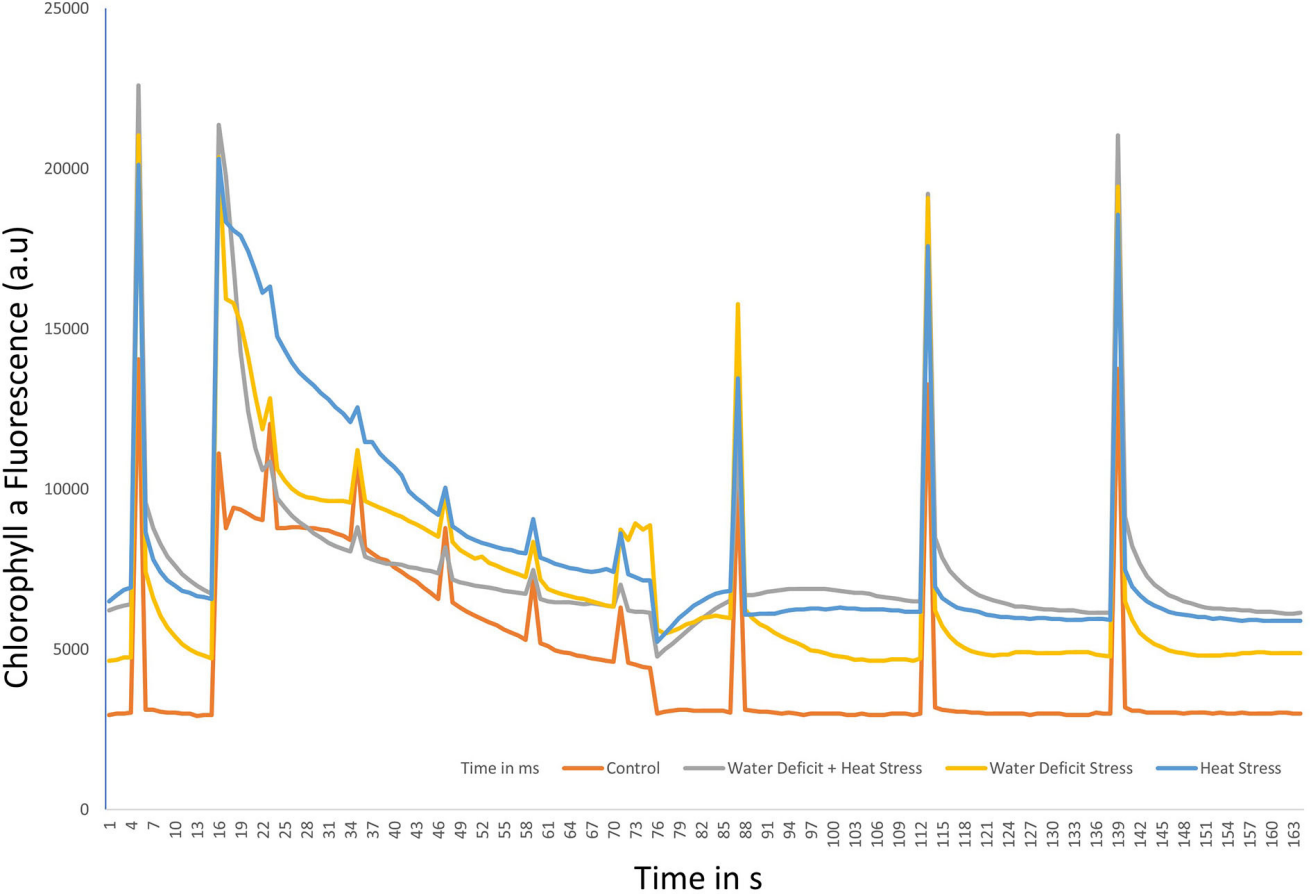
Non-photochemical Quenching (NPQ) under water-deficit stress and heat stress in isolation and in combination in ICMH356 variety pearl millet.

Non-photochemical quenching in the varieties of pearl millet plants under WDS and HS in isolation and in combination for variety ICMH 356 is shown in Figure 5. The heat-stressed plants showed higher NPQ than water-stressed plants and control. There was a general decline observed in the NPQ from approximately 19 s to 76 s. In all the plants, irrespective of the treatments, the NPQ showed a general pattern of five peaks at 4 s at the dark-adapted state maximum fluorescence, which was measured at the first saturation flash after dark adaptation (fm), 16-s fluorescence in the peak of fast Kautsky induction (Fp), 88, 114, and 140 s. The decline from the second peak at 16 s was the longest, with 72 s between peaks of 16 s and 88 s in all the plants. This decline area corresponds to Fm_L, Lss, D, Dss1, where L comprises light-adapted parameters; D represents the dark recovery phase after actinic illumination is switched on; N is a sequential number of the light phase; ss is the steady state. The peaks from 88, 114, and 140 s correspond to the NPQ observed. All the stresses showed high non-photochemical quenching with heat-stressed plants and combined WDS and heat-stressed plants, showing higher values than water stress and control.

Chlorophyll a, chlorophyll b, and the ab ratio and carotenoids are given in Table 3. Chlorophyll a increased in water-stressed plants as compared to control and reduced in the heat-stressed and combined stressed plants. The values in HS recovery and HS, in combination with WDS recovery, were significantly higher than in the corresponding stress treatments. Chlorophyll b, on the other hand, increased significantly in all the stress treatments, exhibiting a progressive decrease in water stress, HS and HS in combination with WDS treatments in that order. The chlorophyll a b ratio followed a general reducing trend, with the only difference that it recovered back to control values as seen in the corresponding stress recovery plants. Total carotenoids increased in all the stress treatments, with a significant increase in HS and water deficit + HS plants as compared to only WDS. Total carotenoids returned to control values in all the corresponding treatment-recovered plants.

**Table 3:** Chlorophyll a, Chlorophyll b, the ab ratio, and total carotenoids in two varieties of pearl millet under water-deficit stress and heat stress in isolation and in combination and after recovery from stress.

**Treatment**	**Chlorophyll a mg**		**Chlorophyll b mg**		**Chlorophyll**		**Total carotenoids mg**	
	**g**^**−1**^ **of fresh weight**		**g**^**−1**^ **of fresh weight**		**a b ratio**		**g**^**−1**^ **of fresh weight**	
	**86M86**	**ICMH356**	**86M86**	**ICMH356**	**86M86**	**ICMH356**	**86M86**	**ICMH356**
Control	1.297a (± 0.032)	1.276a (± 0.032)	1.236a (± 0.04)	1.241a (± 0.004)	1.049a (± 0.019)	1.028 (± 0.006)a	5.96a (± 0.131)	5.36a (± 0.193)
Water deficit stress	1.321b (± 0.02)	1.301b (± 0.06)	1.367b (± 0.05)	1.351b (± 0.006)	0.966b (± 0.04)	0.962 (± 0.009)b	6.79b (± 0.213)	6.72b (± 0.116)
Heat stress	1.213c (± 0.015)	1.223c (± 0.019)	1.301c (± 0.021)	1.321b (± 0.034)	0.932c (± 0.043)	0.925 (± 0.054)c	7.43c (± 0.184)	7.56c (± 0.117)
Water deficit + heat stress	1.202c (± 0.012)	1.22c (± 0.016)	1.301c (± 0.043)	1.311b (± 0.021)	0.923c (± 0.0)	0.930 (± 0.08)c	7.41c (± 0.217)	7.39c (± 0.243)
Control recovery	1.301a (± 0.010)	1.299a (± 0.012)	1.232a (± 0.054)	1.239a (± 0.043)	1.056a (± 0.022)	1.048 (± 0.021)a	5.87a (± 0.179)	5.42a (± 0.121)
Water deficit stress recovery	1.311a (± 0.021)	1.3b (± 0.05)	1.202b (± 0.032)	1.211b (± 0.051)	1.090b (± 0.021)	1.073 (± 0.003)b	6.01a (± 0.213)	5.52a (± 0.197)
Heat stress recovery	1.258d (± 0.091)	1.246d (± 0.0043)	1.276b (± 0.000)b	1.278b (± 0.047)	0.985a (± 0.089)	0.974 (± 0.029)a	6.04a (± 0.122)	6.11a (± 0.264)
Water deficit + heat stress recovery	1.246d (± 0.076)	1.249d (± 0.028)	1.298b (± 0.054)	1.202b (± 0.09)	0.959a (± 0.004)	1.039a (± 0.063)	6.09a (± 0.123)	6.15a (± 0.197)

### PS II electron transport and energy fluxes

**Figure 6:**
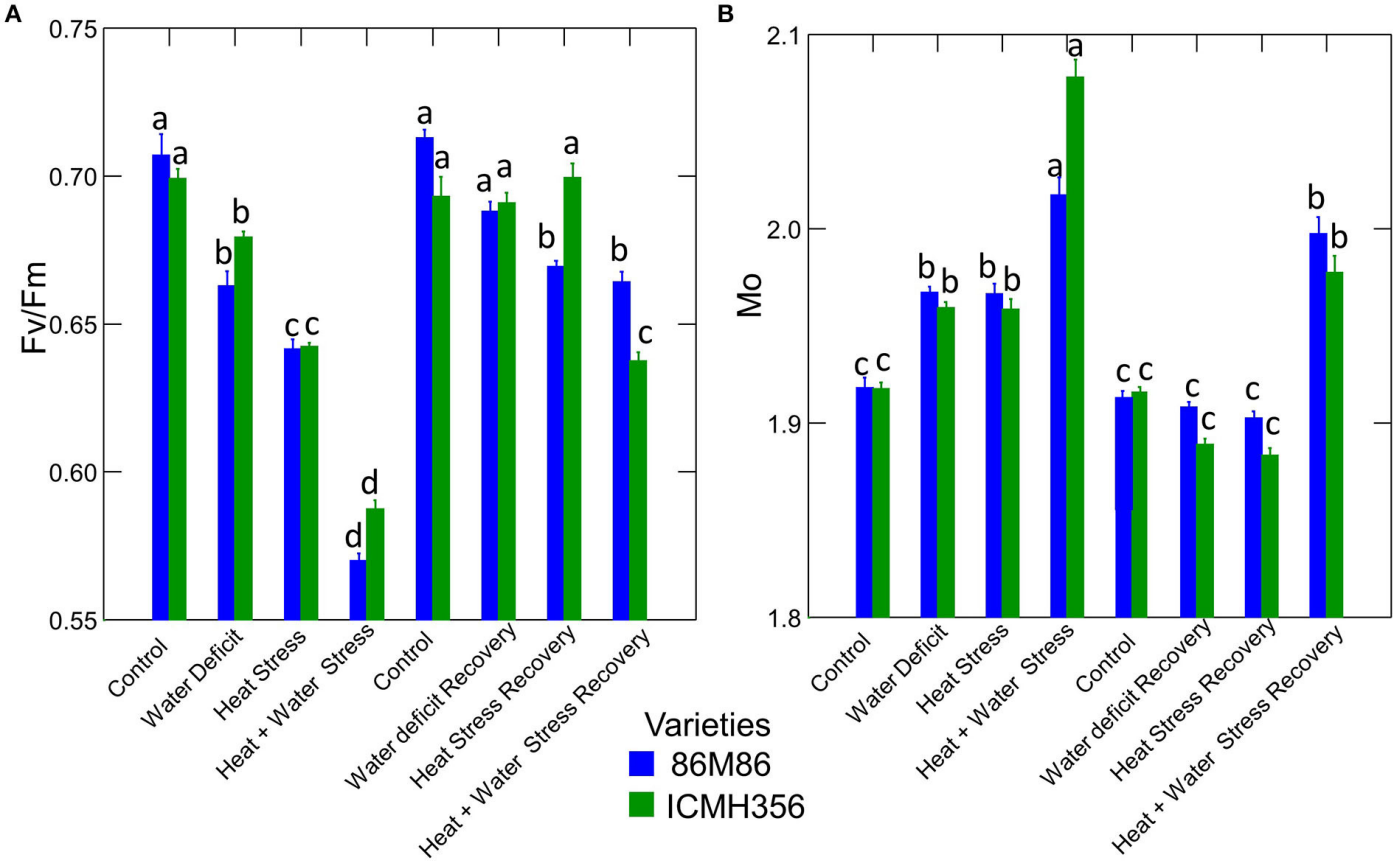
Maximum quantum yield of PSII (Fv/Fm) **(A)** and the Net rate of PS II RC closure (Mo) in two varieties of pearl millet under water-deficit stress and heat stress in isolation and in combination and at recovery from stress **(B)**. Bars represent SE. Letters indicate significance at 0.05, and different letters indicate significant difference between treatments.

The maximum quantum yield of PSII (Fv/Fm) and the net rate of PS II RC closure (Mo) in two varieties of pearl millet under WDS and HS in isolation and in combination and at recovery from stress are given in Figure 6. The maximum quantum yield of PSII decreased significantly in the stress treatments, with each treatment being significantly different from the other. The lowest value of Fv/Fm was seen in the CS treatment. Among the recovery plants, only water-stressed plants were able to recover to levels comparable to control, whereas, in the heat-stressed treatment and the combined stress, the recovery was not full, although there was a considerable increase in the value of Fv/Fm. The variety ICMH 356 showed better HS recovery than the variety 86M86; however, this was not the case in combined stress where 86M86 showed better recovery.

**Figure 7:**
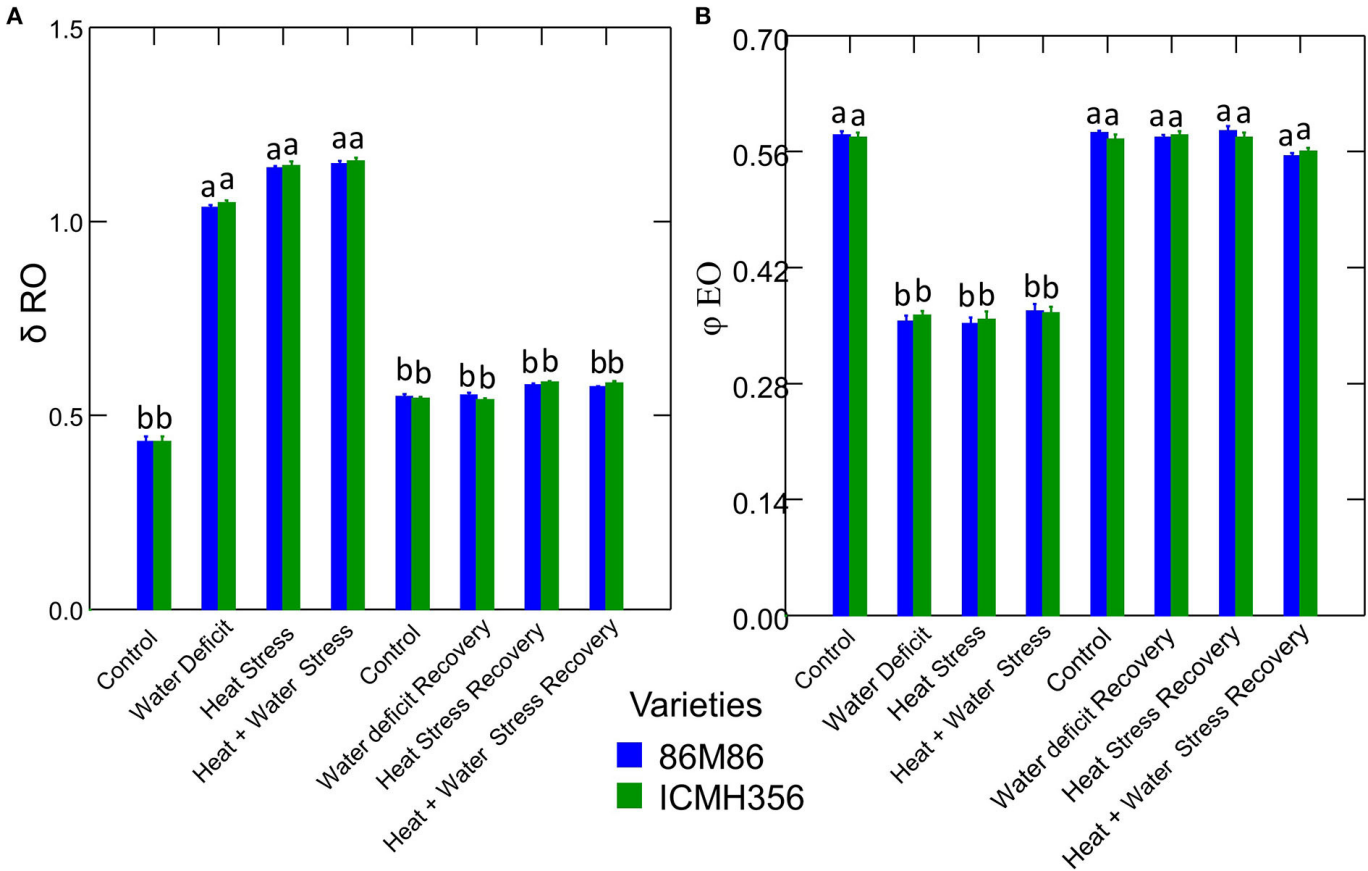
Electron transport efficiency δ RO **(A)** and probability ϕ EO **(B)** parameters in two varieties of pearl millet under water-deficit stress and heat stress in isolation and in combination and at recovery from stress **(B)**. Bars represent SE. Letters indicate significance at 0.05, and different letters indicate significant difference between treatments.

**Figure 8:**
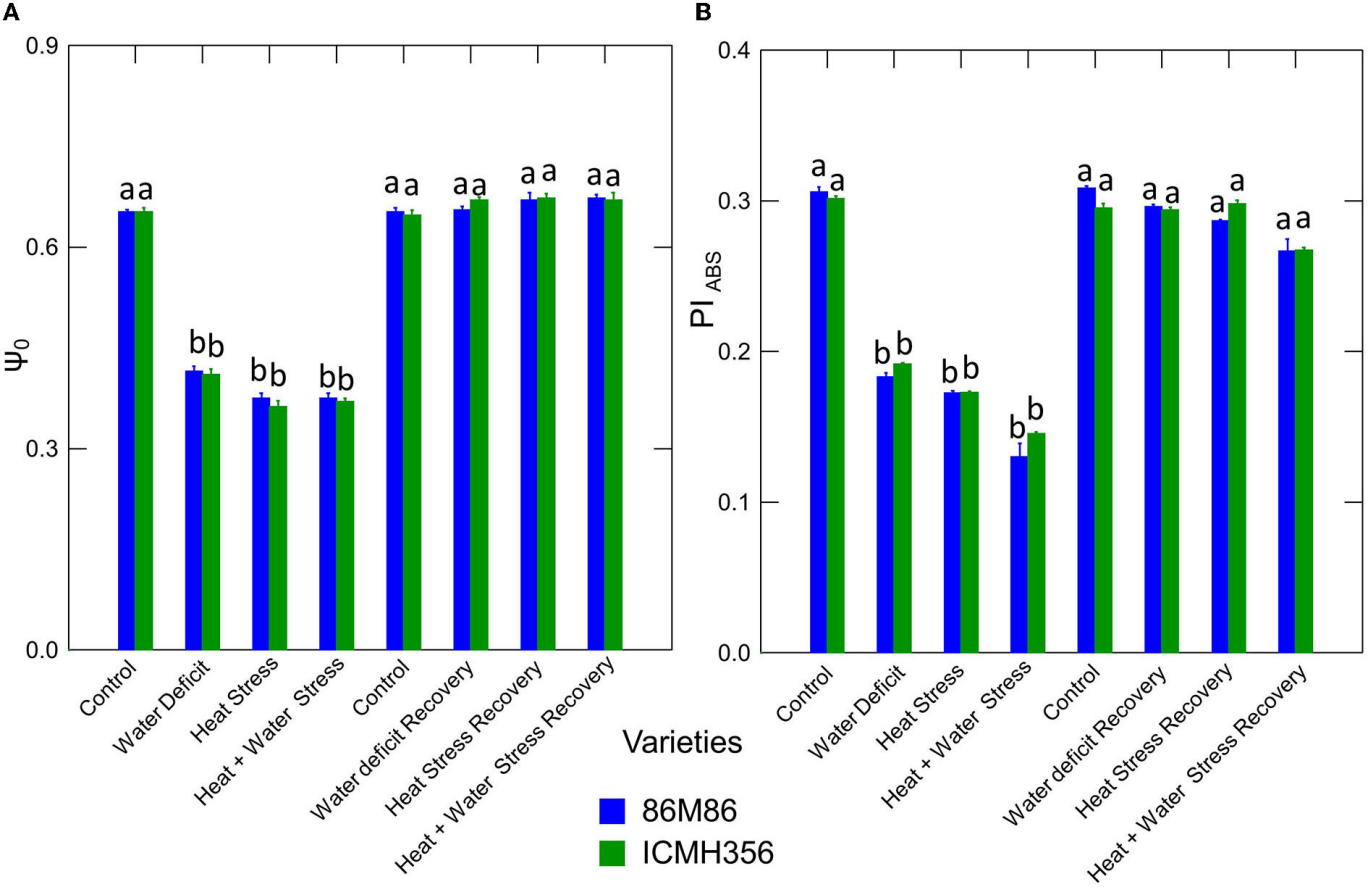
Electron transport progression probability ψ0 **(A)** and Performance Index on an absorption basis (PIABS) **(B)** in two varieties of pearl millet under water-deficit stress and heat stress in isolation and in combination and at recovery from stress **(B)**. Bars represent SE. Letters indicate significance at 0.05, and different letters indicate significant difference between treatments.

The net rate of PS II RC closure (Mo) increased significantly in the stress treatments, with a maximum increase seen in the CS treatment. Similarly, the recovery plants showed the same trend of reversal of the net rate of PS II RC closure with minimum reversal seen in the CS treatment. Electron transport efficiency δ RO and probability ϕ EO in pearl millet plants as affected by WDS and HS in isolation and in combination are shown in Figure 7. The Electron transport efficiency δ RO showed a significant increase in all the stress treatments, and the values came back to levels comparable to control in the respective recovery plants. An opposite trend was seen in the parameter, which indicates the probability of the absorbed photon to move an electron into the electron transport chain (ϕ EO); this probability decreased significantly due to all the stresses and increased and recovered at the recovery phase. Figure 8 shows the probability of a PSII-trapped electron to be transported from QA to QB (ψ0) and the performance index on an absorption basis; both these parameters exhibited a significant decrease due to both WDS and HS in isolation and in combination. Similar to the ϕ EO and δ RO, these parameters were able to recover in the respective stress treatment recovery plants.

**Figure 9:**
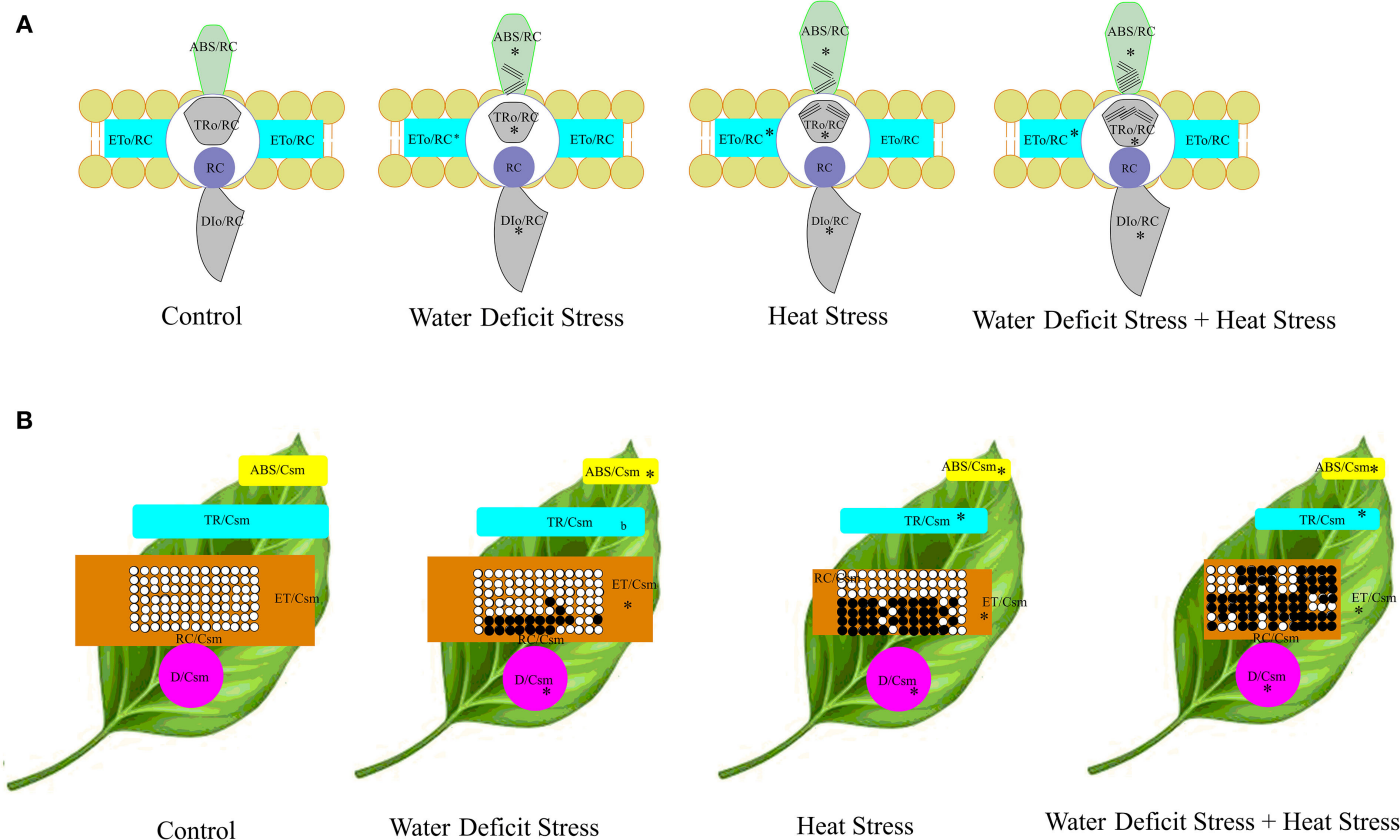
Energy pipeline models of well-watered control and stressed pearl millet plants. The specific energy fluxes per reaction center (RC), which are Absorption (ABS/RC), Trapping flux (TRo/RC), Electron Transport flux (Eto/RC), and Dissipation flux (DIo/RC), are represented in the membrane model as embedded photosystem II proteins or boxes **(A)**. The Phenomenological fluxes per excited cross-section (CS) are shown in the leaf model **(B)**, which are Absorption (ABS/Csm), Trapping flux (TRo/Csm), Electron Transport flux (Eto/Csm), and Dissipation flux (DIo/Csm). In the membrane model **(A)**, the apparent antenna size represents the approximation of the value of absorption per reaction center, which expresses the total absorption flux of the antenna chlorophylls of PS II of both active and inactive centers divided by the active RCs. The inactive centers are represented by the lines on the corresponding embedded proteins on the membrane. Asterisks indicate significance at the 0.05 probability level. In the leaf model, the thickness of the boxes is represented by the values; open circles indicate active reaction centers, and the black circles represent inactive reaction centers.

To understand and elucidate the relationship of the energy fluxes and phenomenological fluxes in a detailed manner, the energy pipe model was constructed both on a membrane basis and a leaf cross-section basis, which is shown in Figure 9. In the membrane model, the specific energy fluxes per reaction center (RC), which are Absorption (ABS/RC), Trapping flux (TRo/RC), Electron Transport flux (Eto/RC), and Dissipation flux (DIo/RC), are represented in the membrane model as embedded photosystem II proteins or boxes showed an increase as seen as bigger boxes or shapes in the stressed plants, with a higher increase seen in the combined stressed plants. In the leaf cross-section model, the Phenomenological fluxes per excited cross-section (CS) are represented as Absorption (ABS/Csm), Trapping flux (TRo/Csm), Electron Transport flux (Eto/Csm), and Dissipation flux (DIo/Csm). There was a decrease in all the parameters represented in this figure due to both the stresses in isolation and in combination. The inactive reaction centers represented by closed boxes in the leaf model increased under stress and were more in the HS treatment and the CS treatment.

## Discussion

### Gas exchange parameters

Our results show that the photosynthetic rate decreased due to stress. It can be seen from the results obtained in the OJIP test that energy and phenomenological fluxes that caused damage to the PS II were the reason for the reduction in the rate of photosynthesis in the stressed plants. The significantly lower photosynthetic rate exhibited by both heat-stressed and water + heat-stressed plants is due to the linear electron transport downregulation in the photosystem II, and this is mainly an adaptive response to circumvent the excessive reduction in the electron transport pathway. The rate of flow of electrons exceeds the rate of CO_2_ assimilation in stress treatments as seen by the decreased photosynthetic rate (Table 2). This inhibition of atmospheric carbon fixation under stress is explained by higher dissipation rates of excess energy, which was also seen in the stressed plants (Figure 5). The increase in transpiration in the HS treatment plants suggests that there was a considerable loss of water in the soil due to a higher temperature regime in the soil in the heat-stressed plant, which is similar to results obtained by Liu and Huang (2005). The increase can be attributed to a mechanism known as evaporative cooling reported by us in a previous study (Shanker et al., 2020). The HSed plants may have a mechanism that triggers the loss of water from the leaf surface at a faster rate to keep the surface cooler where a decrease in the leaf vapor pressure deficit is achieved. The incidence of heat damage to the plants can be minimized by this cooling effect that can result in lower cellular temperatures to maintain optimum metabolism. Stomatal opening to a greater degree in the stressed plants may possibly be the reason for higher stomatal conductance observed. Usually, water stress is mostly accompanied by a degree of temperature increase. In such conditions, the hydraulic conductance in the xylem and mesophyll tissue can increase due to lower water viscosity, and this may explain the stress-dependent opening of the stomata and concomitant increase in the conductance of stomata (Urban et al., 2017).

### Chlorophyll a fluorescence transient kinetics

The JIP test shows the pattern of accumulation of reduced QA, which is inferred as the closure of Reaction Centers (RC) as a result of reduction of QA by PS II proteins and subsequent reoxidation by Photosystem I (PS I) (Strasser et al., 2004). The difference in OJ steps seen in the stressed plants could be due to the reduced reoxidation of QB after the active reduction of QA. The transfer of excitation energy along with the proteins of PS II, which can be termed as PS II connectivity, influenced the OJIP curve. The changes that were seen in the OJIP curve demonstrate that the functional state of photosystem II is sensitive to stress, and differential sensitivity was demonstrated in our study where heat and combined heat and water deficit affected PS II more severely than water-deficit stress in isolation. These findings conform to mathematical explanations offered by Strasser and Stirbet (2001). The disruption of the hyperbolic form of the OJIP curve in the water-deficit-stressed plants and, to a greater extent, in the combined stressed plants suggests that a fraction of PSII with closed centers was more in the stressed plants; this is seen as shown in Figure 9 in the energy pipeline model. The JIP step decrease in stressed plants also indicates that there is a kind of a traffic jam in the electron transport pathway in the donor side of the PS II, with electrons accumulating due to reduced oxidation in the forward steps, thus not allowing electrons to progress in the chain. The exact site of loss of function of PS II and constriction in the electron transport can be ascertained by calculating JIP parameters, such as energy flux, quantum, and phenomenological flux.

### K-step

The imbalances seen in the OJIP steps in the heat-stressed plants were not clearly discernible in the classic OJIP plot. This was specifically for the deviation of relative variable fluorescence (Vt) characteristics, moving away from the typical pattern of the OJIP curve. The most described effect of HS in plants and, also, in many other marine organisms is the decrease in the fluorescence Fm and a concomitant increase in fluorescence F0; this shows the appearance of a peak at 300 μs, which is termed as the K-step (Guisse et al., 1995; Mareckova et al., 2019; Kviderova and Kumar, 2020). The K-step is difficult to discern in the typical OJIP curve (Zivcak et al., 2017); the plot of relative variable fluorescence W = Vt/VJ at 300 μs identified the possible K–step in the plants in our study as affected by HS.

A loss or damage to the OEC has been reported to be the reason for the appearance of the K–step (Zhao et al., 2021; Zhang et al., 2022). There is an active reduction of QA at the acceptor side in the photosystem II when OEC is damaged, and this causes changes in the stable charge separation during this time, which, in turn, causes the appearance of this step. The K-step is hidden in the OJIP curve, specifically at the O-J rise because of the presence of a balance in the electron transport reactions, which cause the rise in fluorescence, so, when there is a deviation in this balance, this K-step arises. Here, in our study, prolonged HS, although not extreme, could have led to the inactivation of the OEC in the heat-stressed and HS in combination with water-deficit-stressed plants. This, possibly, could have caused a disproportionate flow between the electrons emanating from the Reaction Center to the acceptor side and the flow of electrons entering the RC from the donor side of the PS II; this has been shown to be associated with an increased fluorescence yield species thought to be Pheo^−^ acting as a precursor to ${\text{Q}}_{\text{A}}^{-}$ (Strasser et al., 2004). This causes an overall downregulation of the electron transport between OEC and PS II. Some of the explanations for this include electron transport rate limiting at OEC seen as a slower transfer of electrons from water to the amino acid in the D1 protein Tyr161 (Yz) than the speed at which electrons are transferred from Yz to P680+ (Pantazis, 2021). This disruption of OEC by heat in the heat- and water-stressed plants causes an increase in the speed of electron flow forward, the pool of electrons keep moving forward in the chain, and, on the other hand, the supply is exhausted as OEC is damaged. This causes a cascading effect, culminating in lower photosynthetic efficiency and, also, the lower photosynthetic rate as seen in our study (Table 2). A partial loss of manganese cluster has been shown to occur during HS in the OEC (Gupta, 2020; Poor et al., 2021); this can cause dissociation of an Mn-stabilizing enzyme, which is a protein bound on the donor side of the RC of the PS II (Yamane et al., 1998; Zhu Q. et al., 2020). In addition to this, the extrinsic proteins of the PS II, namely, PsbO, PsbP, PsbQ, and PsbR have been shown to disassociate from the OEC complex of PSII (Huang et al., 2020). The K-step could also have been due to restriction of movement in the electron transport chain from Pheophytin to QA and which reflects as structural variations in the LHC of PS II (Plyusnina et al., 2020). In this study, we found that the recovered plants showed the amplitude of the K- step was near to that of control; this may be because OEC damage was reversed during recovery.

The photosynthetic activity as seen by carbon fixation during HS in combination with WDS can be explained by the fact that PSII RCs can access a sizably high alternative pool of electron donors for the fractional reduction of QA and keep the electron transport going even if there is damage to the OEC (Toth et al., 2007). This can also explain the recovery seen in the plants that recovered from heat and combined stress and were able to get back to a near-normal photosynthetic rate.

### Non-photochemical quenching and pigments

NPQ increase in stressed plants suggests that there was triggering of the energy dynamics to dissipate the excess energy by non-radiative decay. It was seen here that the NPQ was stimulated in the stressed plants, and it was at the expense of the process of photochemistry, which was reduced in the stressed plants. The reduction in NPQ was primarily because of the decrease in the light-harvesting antenna size and, also, due to partial closure RCs and limited deactivation of PS II. The dissipation of heat observed in our study in terms of higher NPQ in HS is implicated in the increased de-excitation of the singlet state of chlorophyll in the antenna molecules of PSII. The increase in NPQ in the HS treatments was accompanied by a reduction in the quantum yield of PSII (Figure 6) and an increase in the carotenoid content, which can be due to the effect of xanthophyll reducing the Reactive Oxygen Species (ROS) in the chloroplasts, which can be produced during heat and, also, water stress (Chen et al., 2014). This can be a mechanism by which the plants attempt to maintain photosystem II electron transport homeostasis during stress, and this, in essence, can be an adaptation mechanism and is different from the simple response to stress stimuli like closed-reaction centers. The perturbations in the chlorophyll pigment content and the chlorophyll a b ratios in the stressed plants could be due to upregulation of the xanthophyll cycle in plants to increase the synthesis of zeaxanthin by hydroxylation of β-carotene and further epoxidation of these compounds, leading to the production of neoxanthin (Demmig-Adams and Adams, 1993). The changes in the proportion of the primary light-harvesting pigments could have been due to the diversion of metabolites to the xanthophyll cycle, which helps in adaptation to stress. While the carotenoids play only a small role in the actual gathering of the incident photons and harvesting them and transferring them to chlorophyll molecules, their main role is recognized as protective pigments under stress (Maslova et al., 2021). This increase was clearly seen in the HS treatments in our study. Carotenoids accept singlet oxygen from chlorophyll molecules, which are produced in the chlorophyll under photo and thermal stress (Lee and Min, 1990; Johnson et al., 2022). The increase in carotenoids observed in HS treatments could be due to the role of these pigment complexes in opening rapid channels for quenching of the excited states of chlorophyll *via* quick energy transfer to short-lived excited carotenoid states (Skotnicova et al., 2021).

This quenching function of singlet oxygen of carotenoids can be the reason for the increase in the pigment status in heat-stressed and combination stressed plants. It is possible that the heat-stressed plants are trying to minimize the irreversible damage that can be caused by combination stress. This is evidenced by the fact that plants recovering from heat and water stress in combination regained the near-optimum quantum efficiency, and there was a reduced carotenoid content, which could be due to downregulation of the xanthophyll cycle under no-stress conditions in order to get back to normal rates of CO_2_ fixation. Carotenoids have an essential role in the structural composition of LHC in photosystem II (Peter and Thornber, 2019). The reversal of carotenoid content to control levels may be due to the changes in the structural conformation of LHC II proteins to a non-quenched state.

### Quantum yield, energy, and phenomenological fluxes

The quantum yield of primary photochemistry is represented by the value Fv/Fm. The reduction in the quantum yield of photochemistry was seen in both the water-stressed treatments and HS and combination treatments. The reduction seen in water-stress treatment could have been due to the impairment of the acceptor side of the PS II, leading to the limited reduction of the PQ pool and, further, in the next step, a reduction of the acceptor side of the PS I as seen by results obtained in the other OJIP parameters in the study. The decrease seen in the stress treatments in this value indicates that the yield was affected by both the stresses in isolation and, also, in combination. The reasons for the decrease in Fv/Fm can be possibly attributed to the reduced rate of charge separation, beginning at the very first step of the Z scheme, namely, water splitting. This was evident from the damage to the OEC seen in our study. In addition to this, the stability of the charge separation could also have been affected by a little charge separation that was taking place in spite of the damage to the OEC due to heat (Mathur et al., 2014).

Radical pairs P680 +/Phe– are formed during charge separation (Oxborough and Baker, 2000). The rate constant of recombination of these radical pairs in the RC could have been affected as a consequence of HS resulting in lower quantum yield. One other important reason for the reduction in Fv/Fm could also be from the physical disconnection of some of the antennae from the PS II (Figure 9).

The value Mo gives a quantification of the net rate of closure of PS II as derived from RC trapping minus electron transport. The increase in this parameter in the stress treatments, especially in the heat-stressed plants, indicates impairment of the photosynthetic rate in the stressed plants as seen in our study. The increase higher in HS treatment could be because of increased reduction of QA to Q$\bar{\text{A}}$, resulting in a faster rate of closure of the RC in the HS and the CS treatments as against water stress and control (Gupta, 2019). The heat-stressed and the combined-stressed plants evidently had faster rate closure of RCs This can be viewed as a counteractive measure; in essence, the heat-stressed plants employed a strategy to reduce RC as an adaptation as against increasing the QA reduction rates. This is in tandem with the other results obtained in this study, which shows that there is a reduction in the probability of a trapped electron in PS II traveling on from QA to QB. The possibility that the net rate of closure can keep the light absorption capacity during stress as a counteractive measure to compensate for reduction in the number of RCs is suggested by Duarte et al. (2016); this could well be an HS adaptation in plants. The physiological interpretation of significantly higher Mo in HS treatments and CS treatments is that fractional or partial reduction of QA to QA—in the total pool, QA is higher in HS than in the water-stressed plants.

δ RO is an indication of the efficiency of an electron in the electron transport pathway of the PSII, with the ability to move away from a reduced intersystem electron acceptor. In addition, δ RO plays a role in reducing the electron acceptors present at the end of the transport chain in PSI (Yao et al., 2017; Petrova et al., 2021). Our result in this study suggests that HS in pearl millet increases the probability of an electron being transported to the final electron acceptor in PSI. A higher value of δRo also indicates that the size of the complete pool of end electron acceptors in PSI increases with HS. The dark reaction, which follows the photochemistry, is the next step, so this might indicate that thermal stress could have impaired the dark reaction, and there is an electron stagnation in the PS I due to this as indicated by the value of δRo. The higher degree of inefficiency of the dark reaction can be thought of to correspond to the higher values of δRo in heat stress and combined stress. The recovery indicates that this probability of the electron traveling to the final acceptor is reduced and can be indicative of resumption of optimum rates of dark reaction, and this is supported by the results of photosynthetic rates we obtained in the recovered plants. The recovery in itself was not complete in all the stress treatments with WDS in combination with HS treatment, showing the least recovery. Results similar to this have been reported by Gupta (2019) pigeonpea (*Cajanus cajan* L.) under HS. In addition to this, the increase in the values of δRo also suggests that there may be an improvement in the movement of the electrons *via* cytochrome b6/f to the PSI end electron acceptors. So, the sum of energy flux to the PSI acceptors is maintained in spite of the overexcitation energy pressure in PSII (Li et al., 2015).

ϕEO is the parameter that indicates the probability that an absorbed photon will move an electron into the Electron Transport Chain (ETC). The decrease in this parameter observed in stressed plants clearly indicates an impaired electron transport chain in the PS II, which is reflected in the overall photochemistry. This decrease corresponds to a decrease in the linear electron transport in the PS II under stress. This parameter is also an indication of maximum quantum yield for electron transport beyond QA (Zhu et al., 2018); hence, it was seen here that HS inhibited the movement of electrons in the electron transport pathway beyond QA, resulting in reduced efficiency of photochemistry.

The energy pipeline model depicts the alterations and perturbation in the specific energy fluxes and the phenomenological fluxes in PS II as affected by both the stresses in isolation and in combination. Both the specific energy flux model and the phenomenological flux model developed from the derived values in our study show that WDS and HS in combination have a much stronger effect on the overall electron transport pathway of the PS II in pearl millet plants; there is a differential response to the stresses in isolation and in combination in the plants and both the varieties tested. WDS had moderate effects on the whole of the system; on the other hand, heat and water stress had strong effects with slower and only near-complete recovery. The main action of heat and water stress combination was by closure and deactivation of functional reaction centers, whereas the water stress in isolation showed less deactivation and was more toward partly diminishing the RCs. In combined stress, the overall system was affected in a stronger manner than in the water stress alone; the action of combined stress was seen on all the components of photosystem II, such as the absorbance of photon energy, the trapping of the energy, and the flux and transport of energy.

In the membrane model, the ratio ABS/RC represents the mean size of the antenna part of PSII, and it effectively expresses the absorption potential of the chlorophyll antenna molecules per number of active QA reducing reaction centers (Mathur et al., 2013). The antenna size increase seen in the combination stressed plants indicates that there is more number of active centers. The changes observed in the combination stress plants could also be due to the transition of the PS II units into heat sink units, which is seen in HS in general.

The centers that are mainly from the conversion of QA–reducing centers to non-QA-reducing centers in PS II are called the heat sink units. These heat sink centers form a small portion of PS II reactions centers (RCs) that cannot reduce the primary quinone acceptor of PS II, QA, the so-called silent centers (Xiao et al., 2020). The maximum rate of exciton trapping by the RC, which results in the reduction of QA^−^, is given by the ratio TRo/RC, which shows an increase in the heat-stressed plants, and, this, at the first glance, seems counter-intuitive as the increase in this ratio should increase the electron transport at face value. The explanation for this is that there is a considerable reduction of QA^−^, and, on the other hand, it is not being oxidized back due to inhibition of reoxidation of QA^−^, which is the effect of HS here in the plants. This prevention of reoxidation inhibits the ability of QA^−^ to transfer an electron to QB in an efficient manner. This is corroborated by the fact that we found in our study that ψ0, which represents the probability of a PSII-trapped electron to be transported from QA to QB, declined in stress treatment, with more reduction in heat and combined stress treatments. The energy in the process is lost in dissipation, which we have observed in the increased NPQ observed in the study in the heat and combined stress plants. Results similar to this have been observed in combined stressed plants by Mathur et al. (2013).

Eto/RC represents the amount of reoxidation of the reduced QA^−^ that is taking place in the RC *via* the electron transport pathway in the photosystem II, reflecting only the proportion of active RC as against all the RCs. There was a significant increase in the electron transport for each reaction center as seen in Figure 9. This could be due to the fact that there are more inactive centers, and electron transfer from QA to QB is not efficient and is much less as compared to water-stress-alone treatments. DIo/RC is given by the ratio that throws light on the proportion of total dissipation in the form of heat of the excitation energy that has not been trapped in the system from the total number of RCs in relation to the RCs, which are active. The amount of dissipation would be directly proportional to the ratio of active to inactive RC. The higher the inactive RCs, the higher the dissipation would be, and this was seen in the case of heat-stressed in pearl millet plants. This could have been because of the inability of the inactive centers to effectively trap photons striking on the pigment molecules in the photosystem II, and this causes an increase in untrapped photons and a concomitant increase in dissipation. The flux ratios of plants experiencing combined stress when compared to the plants that were experiencing water stress only were different. Similar results have been reported by Gupta et al. (2021) in Noni (*Morinda citrifolia* L.) plant under temperature stress.

**Figure 10:**
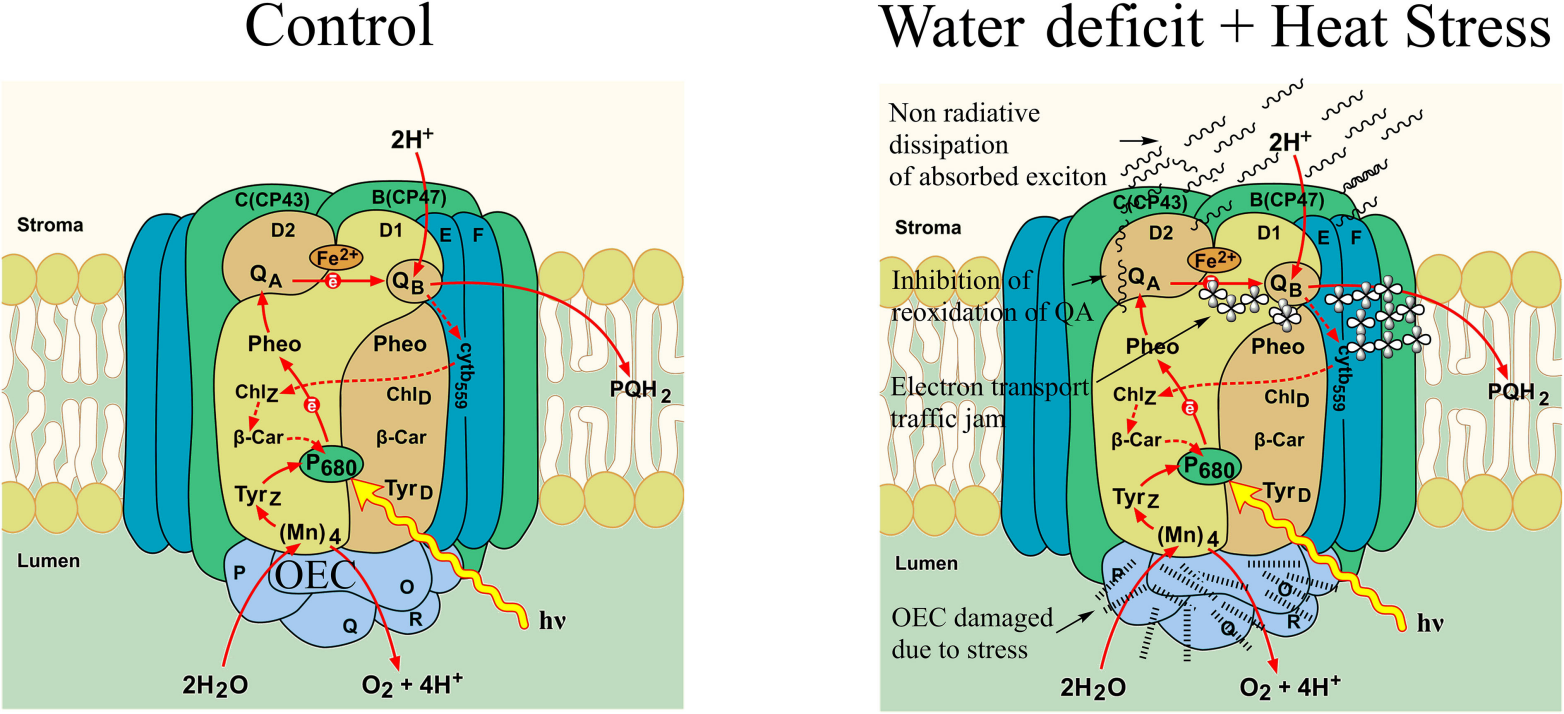
A schematic representation of electron transport in photosystem II in pearl millet as affected by control and water deficit + heat stress.

ABS/CSo is a derived parameter that depicts exciton absorption flux per cross-section of apparent antenna size. The general decrease observed in this parameter suggests lower energy absorbed for every excited cross-section unit area, and this showed that the CS treatments due to the involvement of heat have lower-energy absorption per cross-section mainly because of OEC damage and, also, the impairment of the donor side of the PSII, thus resulting in lower electron flow and creating an electron transport traffic jam in the donor side. The increased reduction in the ETo/CSo in CS treatment could be due to the cascading effect of the lower-energy absorption ratio (ABS/CSo) and, also, lower energy trapping TRo/CSo of the incident photons. Figure 10 shows a schematic representation of electron transport as affected by control and combined stress in pearl millet. In the heat stress-treated plants and the combined stressed plants, the increased number of closed reaction centers could also be due to the same reason, with the OEC damage being one of the initial reasons for the disruption and congested electron flow in the electron transport pathway of the photosystem II. The observed differences in perturbations in the electron transport due water deficit and heat stress reflected correspondingly in the performance index on an absorption basis in the plants. Similar observations have been reported by various studies in other abiotic stresses like salinity, drought, heavy metal, and cold stress (Cicek et al., 2019; Che et al., 2021; Giorio and Sellami, 2021; Janeeshma et al., 2021).

## Conclusion

The understanding of the general and specific mechanism by which higher plants adjust their photosynthetic machinery to water regimes and temperature changes is not an easy task, considering the amount of complexity and interplay involved in this. One of the main reasons for this is that crops exhibit diverse phenology in growth and development; they have a hard-coded genetic potential, which, in itself, is diverse. Most importantly, they respond differently to durational regime changes in water deficit and heat with short-, medium-, and long-term effects being vastly different and, similarly, recovery from this different duration of stresses being equally different.

In our study, we saw some differences in response to and recovery from water deficit and HS in isolation and in combination in the varieties studied. In most of the parameters studied, there was a consistently similar response; some differences were observed. The variety ICMH 356 showed better HS recovery than the variety 86M86; this can be attributed to the stay-green quality of the variety; however, this was not the case in combined stress where 86M86 showed better recovery. It was seen in the study that there was a lowered photosynthetic rate in the stressed plants, which could have contributed to a reduced growth during the stress period. Here, in this study, we bring out the mechanisms by which both heat and water stress in isolation and in combination affect the photosynthetic electron transport in Photosystem II. We show that OEC damage is the main effect of heat stress, which is not seen in the same intensity in the water-stressed plants. We see a distinct differential response to these stresses in the plants and a differential response in recovery. The recovery was not complete in all the stress treatments, with water deficit stress in combination with heat stress treatment showing the least recovery

## Data availability statement

The original contributions presented in the study are included in the article/Supplementary material, further inquiries can be directed to the corresponding author.

## Author contributions

AS: study conception, design, experiment planning, and conducting the experiment. AS, SA, DB, JS, and NJ: recording observations. SA and DB: tabulating data. AS, SA, and DB: analyzing data. AS and JS: data collection. AS and BS: writing. AS: critical revision. SY and MV: data analysis. MV: critical writing. SY, MM, and VS: overall coordination. BS and MM: revision and editing. NJ: experiment conduction. JS: data collection.BS: critical analysis. MM: conceptual help. All authors contributed to the article and approved the submitted version.

## Conflict of interest

The authors declare that the research was conducted in the absence of any commercial or financial relationships that could be construed as a potential conflict of interest.

## Publisher's note

All claims expressed in this article are solely those of the authors and do not necessarily represent those of their affiliated organizations, or those of the publisher, the editors and the reviewers. Any product that may be evaluated in this article, or claim that may be made by its manufacturer, is not guaranteed or endorsed by the publisher.
