# Supplementary material for: High-resolution dissection of photosystem II electron transport reveals differential response to water deficit and heat stress in isolation and combination in pearl millet [Pennisetum glaucum (L.) R. Br.]
Source: Front Plant Sci. 2022 Aug 12;13:892676. doi: 10.3389/fpls.2022.892676 (PMC9412916; doi:10.3389/fpls.2022.892676)
Supplement: Supplementary file 1 [file Table_1.DOCX]

Supplementary Table 1 Soil Moisture Content in Volumetric Water Content (VWC) and Relative Water Content (RWC) of fully expanded leaves in water stress treated pearl millet plants from 0 to12 days after withholding irrigation and 4 days after recovery.

| Days after Treatment | Soil Moisture content  Volumetric Water Content (VWC) | | | Relative Water Content (%) |
| --- | --- | --- | --- | --- |
|  | Soil Depth 7.6 cm | Soil Depth 12 cm | Soil Depth 20 cm |  |
| 0 | 42.1 | 39.8 | 36.2 | 97.65 |
| 2 | 36.9 | 34.2 | 31.8 | 94.12 |
| 4 | 30.1 | 27.2 | 25.1 | 92.12 |
| 6 | 20.2 | 21.7 | 23.8 | 81.12 |
| 8 | 15.3 | 17.5 | 18.4 | 75.23 |
| 10 | 10.6 | 10.2 | 10.9 | 71.12 |
| 12 | 10.2 | 9.9 | 10.6 | 60.34 |
| Recovery at 4 days after start of rewatering | 43.9 | 40.1 | 37.3 |  |
